# Procyanidins Extracted from Lotus Seedpod Ameliorate Amyloid-*β*-Induced Toxicity in Rat Pheochromocytoma Cells

**DOI:** 10.1155/2018/4572893

**Published:** 2018-10-28

**Authors:** Hao Huang, Peipei Yan, Taoping Sun, Xiaoxing Mo, Jiawei Yin, Peiyun Li, Yalun Zhu, Shuang Rong, Wei Yang, Xiaoyi Chen, Liegang Liu

**Affiliations:** ^1^Department of Nutrition and Food Hygiene, Hubei Key Laboratory of Food Nutrition and Safety, Tongji Medical College, Huazhong University of Science and Technology, Wuhan 430030, China; ^2^Ministry of Education Key Lab of Environment and Health, School of Public Health, Tongji Medical College, China; ^3^Chongqing Center for Disease Control and Prevention, Chongqing 400000, China; ^4^Department of Nutrition and Food Hygiene, School of Public Health, Medical College, Wuhan University of Science and Technology, Wuhan 430065, China; ^5^School of Public Health, Guangzhou Medical University, Guangzhou 511436, China

## Abstract

Alzheimer's disease (AD) is a progressive neurodegenerative disease, which is characterized by extracellular senile plaque deposits, intracellular neurofibrillary tangles, and neuronal apoptosis. Amyloid-*β* (A*β*) plays a critical role in AD that may cause oxidative stress and downregulation of CREB/BDNF signaling. Anti-A*β* effect has been discussed as a potential therapeutic strategy for AD. This study aimed to identify the amelioration of procyanidins extracted from lotus seedpod (LSPC) on A*β*-induced damage with associated pathways for AD treatment. Rat pheochromocytoma (PC12) cells incubated with A*β*_25–35_ serve as an A*β* damage model to evaluate the effect of LSPC *in vitro*. Our findings illustrated that LSPC maintained the cellular morphology from deformation and reduced apoptosis rates of cells induced by A*β*_25–35_. The mechanisms of LSPC to protect cells from A*β*-induced damage were based on its regulation of oxidation index and activation of CREB/BDNF signaling, including brain-derived neurotrophic factor (BDNF) and phosphorylation of cAMP-responsive element-binding (CREB), protein kinase B (also known as AKT), and the extracellular signal-regulated kinase (ERK). Of note, by high-performance liquid chromatography-tandem mass spectroscopy (LC-MS/MS), several metabolites were detected to accumulate *in vivo*, part of which could take primary responsibility for the amelioration of A*β*-induced damage on PC12 cells. Taken together, our research elucidated the effect of LSPC on neuroprotection through anti-A*β*, indicating it as a potential pretreatment for Alzheimer's disease.

## 1. Introduction

Alzheimer's disease (AD), a progressive neurodegenerative disease, is characterized by extracellular senile plaque deposits, intracellular neurofibrillary tangles, and neuronal apoptosis. Progressive loss of memory and other cognitive functions are typical symptoms in AD [1]. According to the amyloid hypothesis, amyloid-*β-* (A*β*-) related toxicity and imbalance are cardinal reasons that contribute to synaptic dysfunction and subsequent neurodegeneration in AD [2, 3]. A*β* has been, therefore, suggested as a potential therapeutic target for AD treatment [4].

As similar to other age-related diseases, exorbitant oxidative stress is the fundamental feature of AD since A*β* may lead to oxidative stress and macroautophagy [5]. Oxidative stress induced by A*β* may disorder the membrane ion function and glutamate transporters of synapses, resulting in their dysfunction and degeneration [5]. Antioxidant compounds hence may have a positive effect on the mitigation of A*β*-induced damages. AKT (also known as protein kinase B) and extracellular signal-regulated kinase (ERK) are two key kinases in modulating brain-derived neurotrophic factor (BDNF) transcription by activating phosphorylation of cAMP-responsive element-binding (CREB) [6, 7], both of which could be attenuated by A*β* [8, 9]. BDNF, a pivotal role in learning and memory [10, 11], is downregulated by A*β* in AD [12]. The underlying mechanism of A*β* on CREB/BDNF signaling is possible that A*β* inhibits the activation of AKT and ERK, resulting in decreasing phosphorylation of CREB, the upstream of BDNF [13], and then, attenuating both transcriptions of BDNF mRNA and expression of BDNF protein [14]. Therefore, simulating CREB/BDNF signaling against A*β*-induced damage is a promising therapeutic tactic for AD. CREB activators, BDNF imitators, or flavonoid-high dietary habit have been identified to ameliorate AD [15–17]. BDNF and oxidative stress also have an interactive influence *in vivo* [18, 19] so nature compounds are beneficial for AD treatment, which can modulate oxidative stress and CREB/BDNF signaling.

Lotus has been usually used as a Chinese traditional medicine, including its leaf, embryo loti, and seedpod [20, 21]. Procyanidins, as flavonoids, are highly correlated to learning and memory improvement [22, 23] and exhibit the potential for AD treatment [16, 24]. Procyanidins extracted from the lotus seedpod (LSPC) is the fresh and abundant resource of flavonoids [25]. In age-related mice, LSPC has been reported to enhance the abilities of learning and memory [26, 27]. Consequently, we put forward the assumption that LSPC might display the property of anti-A*β* in AD while there was no definitive evidence for its anti-A*β* toxicity function and its main pathways. LSPC, as a complex mixture, is composed of oligomeric procyanidins and polymeric procyanidins such as epicatechin, procyanidins dimers, and quercetin glucuronide [25] while it was insufficient in research exploring its distribution *in vivo*, which might be conducive to expound its impact.

In this study, we aimed to verify its anti-A*β* effects and protective mechanisms as a promising nature production for AD treatment. We evaluated amelioration of LSPC in A*β*_25–35_-induced damage on rat pheochromocytoma (PC12) cells. CREB/BDNF signaling and antioxidant activity were studied as possible pathways. We used LC-MS/MS to analyze its distribution *in vivo*.

## 2. Materials and Methods

### 2.1. Cells and Reagents

PC12 cells were from Tongji Medical College, Huazhong Science and Technology University. LSPC was provided by Huazhong Agriculture University (China). Cell Counting Kit-8 (CCK-8) was purchased from Dojindo (Japan); anti-BDNF antibody was purchased from Elabscience (China); anti-CREB antibody, anti-phospho-CREB (Ser^133^) antibody, anti-AKT antibody, anti-phospho-AKT (Ser^473^) antibody, anti-ERK1/2 antibody, anti-phospho-ERK1/2 (Thr202/Tyr^204^) antibody, and anti-GAPDH antibody were purchased from cell signaling; LY294002 inhibitor for PI3K and PD98059 inhibitor for ERK1/2 were purchased from Selleckchem; lactate dehydrogenase (LDH), superoxide dismutase (SOD), and malonialdehyde (MDA) were from Nanjing Jiancheng Bioengineering Institute (Nanjing, China); gallic acid was purchased from DRE; procyanidin dimer B (PDB) was purchased from Fluka Co.; epigallocatechin gallate (ECG) was purchased from Chromadex; Annexin V-FITC for flow cytometry was purchased from BestBio; Hoechst staining for apoptosis analysis, BCA protein assay kit, and RIPA lysis solution was purchased from Beyotime; all other reagents were purchased from Sigma.

### 2.2. Cells Culture and Dosages of A*β*_25–35_ and LSPC

PC12 cells were cultured in Roswell Park Memorial Institute (RPMI) 1640 medium with 10% fetal bovine serum in an atmosphere containing 5% CO_2_ at 37°C. To decide an intervention dose of A*β*_25–35,_ we added different doses of A*β*_25–35_ (0, 5, 10, 20, and 40 *μ*M) into cells and incubated for six periods of time (6, 12, 24, 48, and 72 h), respectively. A*β*_25–35_ was prepared by dissolved in sterile PBS and aggregated through incubation at 37°C for 4 days. In order to choose an intervention dose of LSPC, we added six dosages of LSPC (1, 2.5, 5, 10, 20, and 40 *μ*g/mL) into 96-well plates 30 minutes before incubation with A*β*_25–35_ for 24 h or without A*β*_25–35_ for 24 h. Dosages for both A*β*_25–35_ and LSPC were determined through measuring cell viability by CCK-8 according to the instruction. In brief, 10 *μ*L CCK-8 was added to each sample (100 *μ*L) with 2 h incubation under 37°C, and absorbance value of each sample was measured by an enzyme immunoassay analyzer (Bio-Tek, USA) at 570 nm.

### 2.3. PC12 Cells Imaging

After determination of A*β*_25–35_ and LSPC doses, cells were cultured as three groups (PC12 cells, PC12 cells with 20 *μ*M A*β*_25–35_, and PC12 cells with 20 *μ*M A*β*_25–35_ and 10 *μ*g/mL LSPC), which were seeded on 6-well plates at a density of 1 × 10^6^ cells/mL. Cells after treatment were fixed by paraformaldehyde and observed morphology under a microscope (Olympus Corporation, Japan).

### 2.4. Hoechst Staining

Cells were seeded on 6-well plates. After intervention as three groups, each group was washed with PBS twice before 800 *μ*L staining buffer was added and subsequently stained with Hoechst staining solution (5 *μ*L) for 30 min in the dark. Cells were imaged on a fluorescence microscope (Olympus Corporation, Japan). Hoechst staining was executed according to the instructions (Beyotime, China).

### 2.5. Flow Cytometry

Cells seeded on 6-well plates were washed with cold PBS twice. The number of cells was kept closing to 1 × 10^6^/mL in each sample and 400 *μ*L 1 × Annexin V was provided. Each sample was incubated with 5 *μ*L Annexin V-FITC staining for 5 min at 4°C. Then 10 *μ*L propidium iodide (PI) staining was added for 5 min at 4°C. Samples were detected by a flow cytometry (Becton Dickinson, USA) and analyzed by FlowJo software (version 7.6). All procedures were consistent with the instructions (BestBio, China). Cells containing Annexin V-positive staining merely were defined to be in early apoptosis (EA), whereas cells stained with both Annexin V and PI were defined to be in late apoptosis (LA). Total apoptosis (TA) consisted of EA and LA.

### 2.6. Determination of Antioxidant Activity

Cells were divided into six groups (PC12 cells, PC12 cells with 20 *μ*M A*β*_25–35_, PC12 cells with 20 *μ*M A*β*_25–35_ and 5 *μ*g/mL LSPC, PC12 cells with 20 *μ*M A*β*_25–35_ and 10 *μ*g/mL LSPC, and PC12 cells with 20 *μ*M A*β*_25–35_ and 20 *μ*g/mL LSPC). The levels of LDH, T-SOD, and MDA were measured depending on the methods [28] and instructions recommended by Nanjing Jiancheng Bioengineering Institute (Nanjing, China). LDH activity of each sample was detected by an enzyme immunoassay analyzer (Bio-Tek, USA) at 450 nm, and the values were expressed as units per liter. For T-SOD, a BCA protein assay kit (Beyotime, China) was applied to determine the values of proteins expression. The absorbance value of each sample was measured at 570 nm, and the values of T-SOD activity was calculated as units per mg protein. Quantification of MDA was stated as nanomoles per mg protein by testing absorbance values at 532 nm.

### 2.7. Western Blot

Cells were cultured as seven groups (PC12 cells, PC12 cells with 20 *μ*M A*β*_25–35_, PC12 cells added with 10 *μ*g/mL LSPC 30 minutes before incubation with 20 *μ*M A*β*_25–35_, PC12 cells with 10 *μ*M LY294002, PC12 cells added 10 *μ*g/mL LSPC 30 minutes before incubation with 10 *μ*M LY294002, PC12 cells with 30 *μ*M PD98059, and PC12 cells added with 10 *μ*g/mL LSPC 30 minutes before incubation with 30 *μ*M PD98059). After intervention, cells were washed three times using cold PBS. After centrifugation (14000 × rpm, 5 min), each sample was collected excluding the supernatant and lysed in 300 *μ*L lysis buffer for 2 h, following centrifugation for 10 min at 14000 × rpm. The proteins in the supernatant were quantified using the BCA method as above. For blot analysis, samples (20 *μ*L, each) were boiled, separated on 7.5%–12% SDS-PAGE, and transferred to polyvinylidene difluoride (PVDF) membranes. The membranes were hybridized with various antibodies overnight at 4°C, including anti-BDNF antibody (1 : 1000), anti-CREB antibody (1 : 1000), anti-phospho-CREB (Ser^133^) antibody (1 : 1000), anti-AKT antibody (1 : 1000), anti-phospho-AKT (Ser^473^) antibody (1 : 1000), anti-ERK1/2 antibody (1 : 1000), anti-phospho-ERK1/2 (Thr202/Tyr^204^) antibody (1 : 1000), and anti-GAPDH antibody (1 : 3000) as internal standard and then incubated with secondary antibodies for 1 h at room temperature. The images were obtained through a Fluorescence Chemical Imaging Analysis System (Syngene, British). The intensities of the bands were analyzed by the ImageJ software.

### 2.8. Quantitative Real-Time PCR (qRT-PCR)

Total RNA was isolated from cells via RNAiso Plus (TaKaRa, China), and cRNA was extracted using the PrimeScript™ RT reagent Kit (TaKaRa, China), all of which were based on the instructions. qRT-PCR was carried out using the SYBR® *Premix Ex Taq™* (TaKaRa, China) with an ABI 7900HT real-time thermocycler (Applied Biosystems, CA), as previously described [29]. The correlated expressions of genes were calculated by 2^–△△CT^ methods. Primers of specific genes, including BDNF (forward: 5′-AGCAGGCTCTGGAATGATGT-3′; reverse: 5′-GGATTTGAGTGTGGTTCTCCA-3′) and GAPDH (forward: 5′-GCCCAGCAAGGATACTGAGA-3′; reverse: 5′-GGATGGAATTGTGAGGGAGA-3′) as control, were synthesized by Sangon Corp. (Sangon Biotech Co., Ltd., China).

### 2.9. Animals

Fourteen male Sprague-Dawley rats (226 ± 35 g, obtained from the Experimental Animal Center of Tongji Medical College, Huazhong Science and Technology University) with two or three per cage were kept in a controlled temperature (23 ± 1°C) under a 12 h dark-light cycle. All rats were free access to deionized water and diet for 1 week. All procedures were in accordance with the guidelines of Tongji Medical College Council on Animal Care Committee, Huazhong Science and Technology University (IACUC number: S407, approval date was 28 March 2015).

### 2.10. LSPC Treatment

Prior to administration of LSPC, rats were randomly divided into two groups (*n* = 7 per group) and fasted for 12 h but had access to deionized water. For the control group, physiological saline was given by oral gavage daily; for LSPC group, LSPC (a brownish red power) was dissolved in physiological saline (20 mg/mL) and administered to rats at a dose of 200 mg/kg body weight by oral gavage daily for two weeks. Body weights were measured every two days. Rats were sacrificed two hours later after a final dose. Tissues (brain, cardiac, liver, kidney, spleen, and pancreas), intestine content, and plasma were harvested and stored at −80°C until analysis.

### 2.11. LC-MS/MS

For the extraction of LSPC and its metabolites, tissues (60 mg) were homogenized with 300 *μ*L mixture (50 *μ*L 1% (*w*/*v*) aqueous ascorbic solution and 250 *μ*L 0.1% formic acid). Ethyl gallate was an internal standard. Each sample was hydrolyzed with a *β*-glucuronidase/sulfatase type H1 (1500 U/mL) from H. pomatia (Sigma, USA) for two hours at 37°C. Then, methanol (200 *μ*L) was added to each sample followed by vibration (30 s) and centrifugation (12000 rpm, 10 min, 4°C), and the supernatant was collected. The extraction was repeated once. The combined supernatants were evaporated to dryness under vacuum at 35°C. The residue was reconstituted in 50 *μ*L of solvent (methanol/water, 1 : 1, *v*/*v*) for LC-MS/MS analysis.

The analysis was performed on a high-performance liquid chromatography-tandem mass spectroscopy (LC-MS/MS, AB Sciex QTrap 4500, Applied Biosystems, Foster City, CA, USA). This method was in accordance with the reported studies [30–32]. Briefly, 5 *μ*L samples were injected for LC-MS/MS, and the analytes were separated by BETASIL Phenyl Column (2.1 mm × 150 mm, 3 *μ*m; Thermo Scientific, USA) at 35°C. The mobile phases composed (a) water with 0.2% acetic acid and (b) methanol with 0.2% acetic acid. Ionization was carried out by electrospray in the negative mode. The calibration curves of respective standards were utilized to quantify compounds. Transition ions, retention times, and mass-spectrometry parameters for all compounds were shown in Table S1; chemical structures of all compounds were exhibited in Figure S1.

### 2.12. Statistical Analysis

The data are presented as mean values ± standard error of the mean (SEM) and analyzed by ANOVA with Student-Newman-Keuls (SNK) or student *t*-test on SPSS software version 19.0. The level of significance was set for *P* value *<* 0.05.

## 3. Results

### 3.1. Dosages of A*β*_25–35_ and LSPC

**Figure 1:**
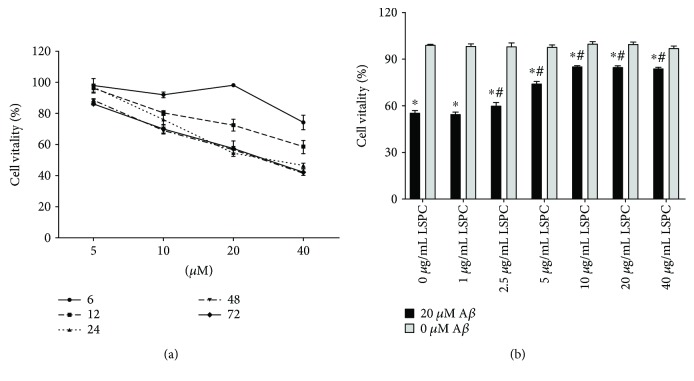
Determination for both A*β*_25–35_ and LSPC through measuring survival rates of cells by CCK-8. (a) Survival rate of cells under variable dosage of A*β*_25–35_ with different intervene time ($\bar{x}\pm SEM$, *n* = 6, %). (b) Survival rates of cells under different dosages of LSPC with 20 *μ*M A*β*_25–35_ (black) and without A*β*_25–35_ (gray) ($\bar{x}\pm SEM$, *n* = 6, %). 0 *μ*g/mL LSPC, PC12 cells with 20 *μ*M A*β*_25–35_ group, and without A*β*_25–35_; 1 *μ*g/mL LSPC, PC12 cells with 1 *μ*g/mL LSPC for 30 minutes before incubation with 20 *μ*M A*β*_25–35_ group, and without A*β*_25–35_; 2.5 *μ*g/mL LSPC, PC12 cells with 2.5 *μ*g/mL LSPC for 30 minutes before incubation with 20 *μ*M A*β*_25–35_ group, and without A*β*_25–35_; 5 *μ*g/mL LSPC, PC12 cells with 5 *μ*g/mL LSPC for 30 minutes before incubation with 20 *μ*M A*β*_25–35_ group, and without A*β*_25–35_; 10 *μ*g/mL LSPC, PC12 cells with 10 *μ*g/mL LSPC for 30 minutes before incubation with 20 *μ*M A*β*_25–35_ group, and without A*β*_25–35_; 20 *μ*g/mL LSPC, PC12 cells with 20 *μ*g/mL LSPC for 30 minutes before incubation with 20 *μ*M A*β*_25–35_ group and without A*β*_25–35_; 40 *μ*g/mL LSPC, PC12 cells with 40 *μ*g/mL LSPC for 30 minutes before incubation with 20 *μ*M A*β*_25–35_ group and without A*β*_25–35_; ^∗^ *P* < 0.05 for groups vs PC12 cells without A*β*_25–35_ and LSPC; ^#^ *P* < 0.05 for groups vs PC12 cells with A*β*_25–35_ but without LSPC. All the results above are the representative of the three independent experiments.

Figure 1(a) demonstrated that 20 *μ*M A*β*_25–35_ had a significant effect on the survival rate of PC12 cells after 24 h intervention that was consistent with the previous report [33]. Thus, we chose the dosage of 20 *μ*M A*β*_25–35_ with the intervention period of 24 h on PC12 cells for further study. In order to testify a dose-dependent manner of LSPC, we added 1, 2.5, 5, 10, 20, and 40 *μ*g/mL LSPC into PC12 cells before A*β*_25–35_ intervention, respectively. As shown in Figure 1(b), the survival rates of PC12 cells under the damage of 20 *μ*M A*β*_25–35_ were gradually improved following the increasing dosages of LSPC until it reached 10 *μ*g/mL. Moreover, there was no toxicity *in vitro* for any dosage of LSPC. 10 *μ*g/mL LSPC was chosen for further study since it exhibited the strongest protection on PC12 cells against the damage harvested from 20 *μ*M A*β*_25–35_.

### 3.2. LSPC Inhibit A*β*_25–35_-Induced Morphology Changes and Apoptosis on PC12 Cells

**Figure 2:**
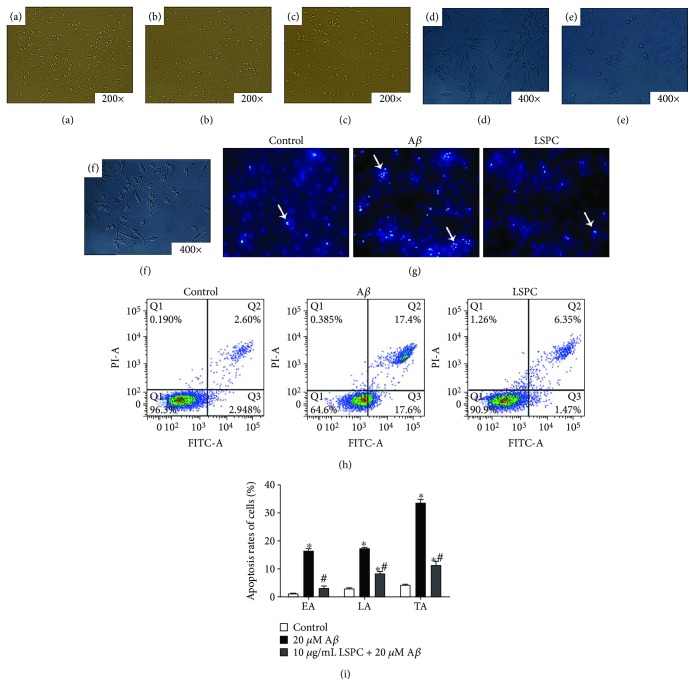
The effect of LSPC against A*β*_25–35_-induced apoptosis on PC12 cells. (a–f) Cellular morphology of different groups under an electron microscope. (a) Control (200×); (b) control (400×); (c) A*β* (200×); (d) A*β* (400×); (e) LSPC (200×); (f) LSPC (400×). Control, PC12 cells; A*β*, PC12 cells with 20 *μ*M A*β*_25–35_; LSPC, PC12 cells added into 10 *μ*g/mL LSPC 30 minutes before incubation with 20 *μ*M A*β*_25–35_. In the electron microscope, PC12 cells with 10 *μ*g/mL LSPC and 20 *μ*M A*β*_25–35_ (e, f) showed the approximate number of cells and identical cellular morphology as a control group (a, b), while A*β* group (c, d) illustrated the reduction of cells number and abnormal morphology. (g) Hoechst staining reflects the apoptosis of cells in each group; apoptosis cells are pointed out by white arrows. (h and i) Flow cytometry calculates apoptosis rates of different groups after Annexin V/PI staining. Apoptosis rate of each group are mean ± SEM; ^∗^ *P* < 0.05 for groups vs control; ^#^ *P* < 0.05 for groups vs A*β*_25–35_ group. Control, PC12 cells; A*β*, PC12 cells with 20 *μ*M A*β*_25–35_; LSPC, PC12 cells added into 10 *μ*g/mL LSPC 30 minutes before incubation with 20 *μ*M A*β*_25–35_. All the results above are the representative of the three independent experiments run in quadruplicate.

Cells were cultured in three groups: control group, PC12 cells with 20 *μ*M A*β*_25–35_, and PC12 cells were added 10 *μ*g/mL LSPC 30 minutes before incubation with 20 *μ*M A*β*_25–35_. In electron microscope (Figures 2(a)–2(f)), PC12 cells with 10 *μ*g/mL LSPC and 20 *μ*M A*β*_25–35_ showed the approximate number of cells and identical cellular morphology as control group, while PC12 cells with 20 *μ*M A*β*_25–35_ exhibited decreased cells number as well as abnormal morphology that PC12 cells shortened and shrank. As Hoechst staining (Figure 2(g)) demonstrated, PC12 cells in 20 *μ*M A*β*_25–35_ group suggested conspicuous karyopyknosis and cell apoptosis compared to control group while addition of 10 *μ*g/mL LSPC prevented the damage from A*β*_25–35_ remarkably. In flow cytometry analysis (Figures 2(h) and 2(i)), we further validated that the apoptosis rates of PC12 cells with 20 *μ*M A*β*_25–35_, including early apoptosis rates (AE), later apoptosis rates (LA), and total apoptosis rates (TA), were higher than that in the control (*P* < 0.05), and addition of 10 *μ*g/mL LSPC significantly lessened apoptosis rates augmented by A*β*_25–35_ (*P* < 0.05).

### 3.3. LSPC Protect PC12 Cells from A*β*_25–35_-Induced Oxidative Stress

**Figure 3:**
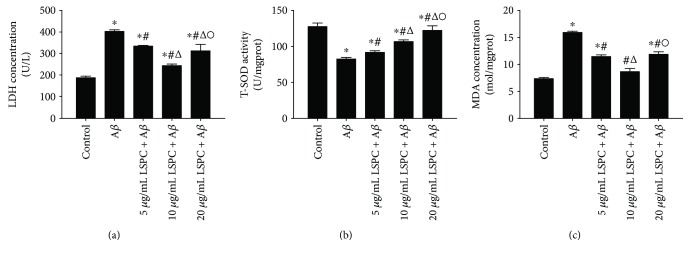
Oxidative index of different groups ($\bar{x}\pm SEM$, *n* = 6, %). (a–c) The LDH concentration, T-SOD activity, and MDA concentration of different groups, respectively. Control, PC12 cells; A*β*, PC12 cells with 20 *μ*M A*β*_25–35_; 5 *μ*g/mL LSPC + A*β*, PC12 cells added into 5 *μ*g/mL LSPC 30 minutes before incubation with 20 *μ*M A*β*_25–35_; 10 *μ*g/mL LSPC + A*β*, PC12 cells added into 10 *μ*g/mL LSPC 30 minutes before incubation with 20 *μ*M A*β*_25–35_; 20 *μ*g/mL LSPC + A*β*, PC12 cells added into 20 *μ*g/mL LSPC 30 minutes before incubation with 20 *μ*M A*β*_25–35._ All data are mean ± SEM. ^∗^ *P* < 0.05 for groups vs control; ^#^ *P* < 0.05 for groups vs A*β* group; ^△^ *P* < 0.05 for groups vs 5 *μ*g/mL LSPC+20 *μ*M A*β*_25–35_; ^o^ *P* < 0.05 for groups vs 10 *μ*g/mL LSPC+20 *μ*M A*β*_25–35_. All the results above are the representative of the three independent experiments.

The antioxidant activity of LSPC against the A*β*_25–35_-induced damage on PC12 cells was determined by evaluating levels of LDH, MDA, and T-SOD. As shown in Figures 3(a)–3(c), compared to control group, PC12 cells with 20 *μ*M A*β*_25–35_ had higher levels of intracellular MDA (*P* < 0.05) and extracellular LDH (*P* < 0.05) and a lower enzyme activity of T-SOD (*P* < 0.05). 5, 10, and 20 *μ*g/mL LSPC all exhibited antioxidant activity. 10 *μ*g/mL LSPC significantly reduced the levels of MDA and LDH among all groups and improved the activity of T-SOD on PC12 cells.

### 3.4. LSPC Ameliorate A*β*_25–35_-Induced Downregulation of CREB/BDNF Signaling in PC12 Cells

**Figure 4:**
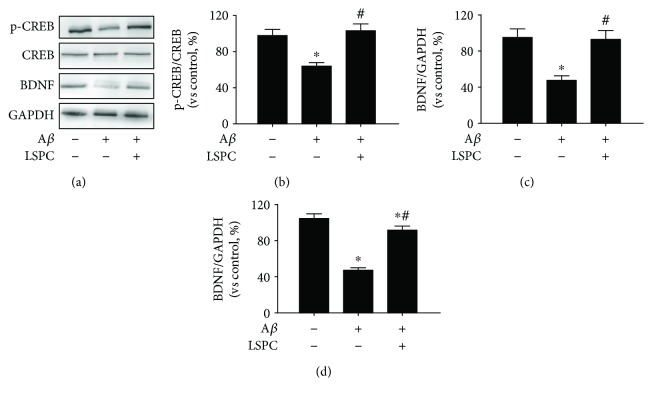
CREB/BDNF proteins expressions and mRNA expressions of intracellular BDNF in three groups (% of control, *n* = 4). (a) CREB/BDNF proteins expressions by Western blotting; (b) p-CREB/CREB proteins expressions by Western blotting in (a) (each group vs control, %); (c) BDNF protein expressions by Western blotting in (a) (each group vs control, %). Control, PC cells; A*β*, PC12 cells with 20 *μ*M A*β*_25–35_ group; LSPC, PC12 cells with 10 *μ*g/mL LSPC and 20 *μ*M A*β*_25–35_ group. All data are mean ± SEM. ^∗^ *P* < 0.05 for groups vs control group; ^#^ *P* < 0.05 for groups vs A*β* groups. (d) mRNA expression of intracellular BDNF in each group by qRT-PCR analysis. Cells were cultured as three groups as above, including control, A*β*, and LSPC. All data are mean ± SEM. ^∗^ *P* < 0.05 for groups vs control groups; ^#^ *P* < 0.05 for groups vs A*β* groups. All the results above are the representative of the three independent experiments.

To substantiate the alleviation effect by LSPC on A*β*_25–35_-induced damage via CREB/BDNF signaling, we employed three groups: control group, PC12 cells with 20 *μ*M A*β*_25–35_ (A*β* group), and PC12 cells with 20 *μ*M A*β*_25–35_ after incubation with 10 *μ*g/mL LSPC for 30 minutes (LSPC group). There was a significant discrepancy in p-CREB/CREB and BDNF expressions between the control group and A*β* group (*P* < 0.05) (Figure 4). LSPC promoted phosphorylation of CREB (Figures 4(a) and 4(b)) and augmented BDNF expression (Figures 4(a) and 4(c)), indicating that LSPC could mitigate A*β*_25–35_-induced diminishment of CREB phosphorylation and BDNF expression. qRT-PCR analysis of BDNF mRNA (Figure 4(d)) demonstrated that A*β*_25–35_ significantly attenuated BDNF mRNA expression compared with control group (*P* < 0.05) while LSPC counteracted the effect of A*β*_25–35_ on BDNF mRNA expression.

**Figure 5:**
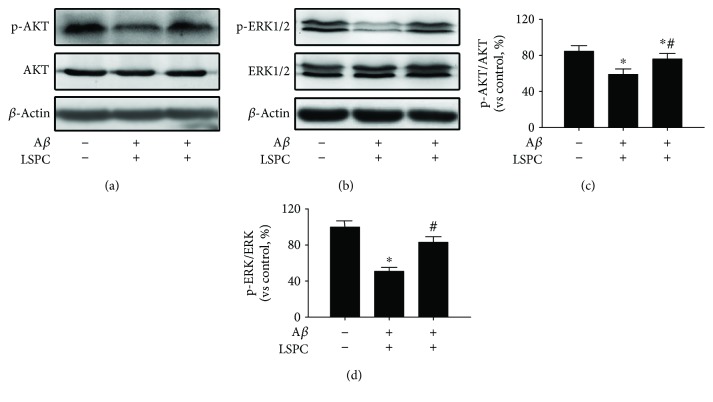
p-AKT/AKT and p-ERK/ERK proteins in each group (% of control, *n* = 4). (a) p-AKT/AKT proteins expressions by Western blotting; (b) p-ERK/ERK proteins expressions by Western blotting; (c) p-AKT/AKT proteins expressions by western blotting in (a) (each group vs control, %); (d) p-ERK/ERK proteins expressions by Western blotting in (b) (each group vs control, %). Control, PC cells; A*β*, PC12 cells with 20 *μ*M A*β*_25–35_ group; LSPC, PC12 cells with 10 *μ*g/mL LSPC and 20 *μ*M A*β*_25–35_ group. All data are mean ± SEM. ^∗^ *P* < 0.05 for groups vs control groups; ^#^ *P* < 0.05 for groups vs A*β* groups. All the results above are the representative of the three independent experiments.

Upstream signaling of CREB/BDNF including PI3K/AKT and Raf/ERK1/2 were analyzed through Western blotting (Figure 5). Both AKT and ERK phosphorylation were diminished after A*β*_25–35_ treatment. LSPC could conspicuously reverse the effects induced by A*β* (*P* < 0.05).

**Figure 6:**
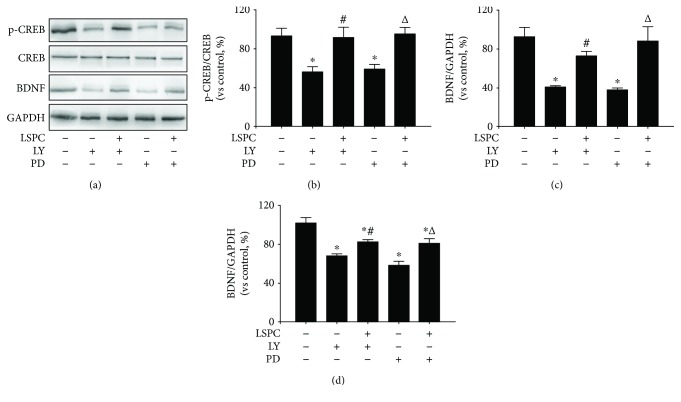
CREB/BDNF proteins expressions and mRNA expression of intracellular BDNF in five groups (% of control, *n* = 4). (a) CREB/BDNF proteins expressions by Western blotting; (b) p-CREB/CREB proteins expressions by Western blotting in (a) (each group vs control, %); (c) BDNF protein expressions by Western blotting in (a) (each group vs control, %). Control, PC cells; LY, PC12 cells with 10 *μ*M LY294002; LSPC + LY, PC12 cells with 10 *μ*g/mL LSPC and 10 *μ*M LY294002; PD, PC12 cells with 30 *μ*M PD98059; LSPC+PD, PC12 cells with 10 *μ*g/mL LSPC and 30 *μ*M PD98059. All data are mean ± SEM. ^∗^ *P* < 0.05 for groups vs control groups; ^#^ *P* < 0.05 for groups vs LY groups; ^△^ *P* < 0.05 for groups vs PD groups. (d) mRNA expression of intracellular BDNF in each group by qRT-PCR analysis. Cells were cultured as five groups as above, including control, LY, LSPC + LY, PD, and LSP + PD. All data are mean ± SEM. ^∗^ *P* < 0.05 for groups vs control group; ^#^ *P* < 0.05 for groups vs LY groups; ^△^ *P* < 0.05 for groups vs PD groups. All the results above are the representative of the three independent experiments.

**Figure 7:**
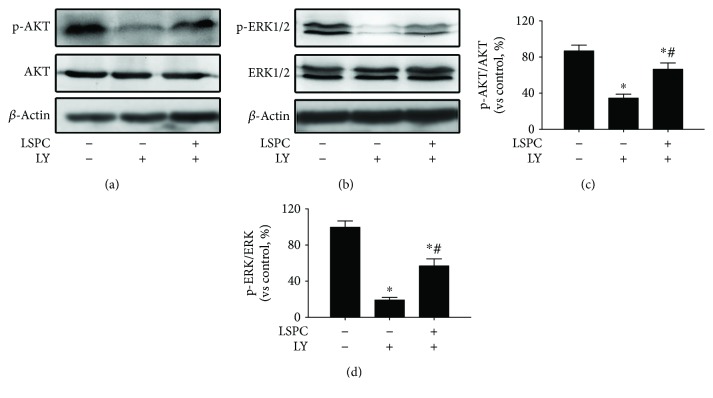
p-AKT/AKT and p-ERK/ERK proteins in each group (% of control, *n* = 4). (a) p-AKT/AKT proteins expressions by Western blotting; (b) p-ERK/ERK proteins expressions by Western blotting; (c) p-AKT/AKT proteins expressions by Western blotting in (a) (each group vs control, %); (d) p-ERK/ERK proteins expressions by western blotting in (b) (each group vs control, %). Control, PC cells; LY, PC12 cells with 10 *μ*M LY294002; LSPC + LY, PC12 cells with 10 *μ*g/mL LSPC and 10 *μ*M LY294002; PD, PC12 cells with 30 *μ*M PD98059; LSPC + PD, PC12 cells with 10 *μ*g/mL LSPC and 30 *μ*M PD98059. All data are mean ± SEM. ^∗^ *P* < 0.05 for groups vs control groups; ^#^ *P* < 0.05 for groups vs LY or PD groups. All the results above are the representative of the three independent experiments.

To further identify CREB/BDNF signaling in neuroprotection of LSPC, we applied LY294002, an inhibitor of the PI3K/AKT pathway, and PD98059, an inhibitor of the ERK pathway. Cells were cultured as five groups: PC12 cells, PC12 cells with 10 *μ*M LY294002, PC12 cells with 10 *μ*g/mL LSPC for 30 minutes before incubation with 10 *μ*M LY294002, PC12 cells with 30 *μ*M PD98059, PC12 cells with 10 *μ*g/mL LSPC for 30 minutes before incubation with 30 *μ*M PD98059. In Figures 6(a)–6(c), LY294002 and PD98059 inhibited the expression of phosphorylation of CREB and BDNF while LSPC reversed the inhibition of LY294002 and PD98059 significantly (*P* < 0.05). In a qRT-PCR analysis of BDNF mRNA (Figure 6(d)), LY294002 and PD98059 notably lessened BDNF mRNA expression and LSPC enhanced BDNF mRNA expression in PC12 cells. Additionally, LSPC counteracted the reduction of AKT and ERK phosphorylation after LY294002 or PD98059 intervention (Figure 7).

### 3.5. Distribution of LSPC and Its Metabolites in Rat Tissues

As Tables 1(a) and 1(b), and 2 illustrated, after two weeks of consecutive LSPC administration, the quantities and formations of compounds varied in rat tissues and plasma. Summarily, PDB, epigallocatechin (EGC), and ECG were not detected in any rat tissues; syringic acid (1.98 ± 0.34) was slightly presented in plasma. After enzyme incubation, there were statistically significant differences (*P* < 0.05) about ferulic acid in pancreas and plasma, m-coumaric acid in the brain tissue, pancreas, and plasma, and protocatechuic acid (PCC) in the brain tissue and plasma.

In brain (Table 1(a) and 1(b)), the quantities of quercetin, epicatechin, gallic acid, vanillic acid, m-coumaric acid, protocatechuic, 3-hydroxyphenylacetic acid (3-HPAA), and pyrocatechol significantly accrued in LSPC group. The enhancement of four compounds was verified after enzyme preprocess with LSPC intervention, these being quercetin, epicatechin, caffeic acid, and 3-HPAA, in both cardiac and liver. Besides, catechin accumulated in the cardiac tissues due to LSPC treatment; in the liver, homovanillic acid (HVA), gallic acid, and 3-hydroxybenzoic acid (3-HBA) increased markedly. Diverse compounds in the kidney (Table 1(a) and 1(b)) indicated significant differences between control and LSPC groups after enzyme disposal, including quercetin, catechin, epicatechin, HVA, caffeic acid, vanillic acid, 3,4-dihydroxyphenylacetic acid (3, 4-DHPA), 3-HBA, and pyrocatechol. In the pancreas (Table 1(a) and 1(b)), there was a remarkable increase in quercetin, ferulic acid, gallic acid, and m-coumaric acid, resulting from LSPC treatment. Apart from quercetin, 3, 4-DHPA alone in the spleen was confirmed to be significantly accrued after enzymolysis. LSPC administration contributed to the accumulations of quercetin, epicatechin, ferulic acid, HVA, caffeic acid, vanillic acid, 3-HBA, syringic acid, p-HPPA, m-coumaric, PCC, and pyrocatechol in plasma (Table 2).

There was no significant difference in body weight between the control group and LSPC group after LSPC treatment (Figure S2). Catechin and epicatechin were distinguished by LC-MS/MS according to distinctive retention time and transition ions.

## 4. Discussion

Recently, there has been an increasing interest in the discovery of potential flavonoids for preventing dementia or AD; nevertheless, the complexity and diversity of flavonoids restrict the understanding of their value on AD treatment. This study comprehensively verified its anti-A*β* neurotoxicity *in vitro* that could alleviate AD-related symptoms.

In AD, A*β* may contribute to oxidative stress in the brain [1, 34] while the antioxidant activity is an outstanding feature of flavonoids. PC12 cells with A*β*_25–35_, as an AD-like model, were performed to testify the abilities about anti-A*β* neurotoxicity of LSPC [35, 36]. LSPC has no toxicity *in vitro* and *in vivo* that coincided with previous studies [27, 37]. LSPC has exhibited its antioxidation effect *in vitro* that was consistent with Xu et al. [27]. Interestingly, a higher concentration of LSPC (20 mg/L) seemed to be less efficient in the decrease of MDA and LDH, and a dose-response could be seen regarding the SOD activity. This result could be partly due to the difference in antioxidant activity associated with doses of procyanidins, cell type, and time of exposure [38]. The inconsistency of different antioxidant enzymes activities has been reported by Puiggròs et al. [39]. Antioxidant reactions of flavonoids, as illustrated by many studies, may benefit the treatment and precaution of cancer [40], cardiovascular diseases [41, 42], type 2 diabetes [41, 42], and neurodegenerative diseases [43]. Since periphery anti-A*β* has been proposed as potential approaches to ameliorate impairment of A*β* [44, 45] in the central nervous system that the liver and kidney have been tightly related to it [44, 46], it is a high possibility that antioxidant effect of LSPC could contribute to alleviate A*β* toxicity in this pathway.

Not only oxidative stress is attributed to accumulation and neurotoxicity of A*β* in AD but also downregulation of CREB/BDNF signaling [5, 12–14]. Several studies have shed the light on anti-A*β* effect of flavonoids [47–49]. Lin et al. have reported that A*β* could induce the death of cells [50], and in AD, it is a major damage resulted from A*β* aggregation [51]. According to Hoechst staining and flow cytometry in the present study, LSPC kept cellular morphology from deformation and suppressed the apoptosis of cells induced by A*β*. In addition, A*β* can reduce the expression of BDNF in AD [52], and CREB can mediate A*β*-induced BDNF downregulation [53] that are in accordance with our results. CREB/BDNF signaling was downregulated by A*β* but upregulated by LSPC. Through targeting phosphorylation of CREB, AKT, and ERK, the upstream of CREB/BDNF signaling can affect BDNF transcription [6, 7]. Activations of both AKT and ERK were restrained by A*β* [8, 9] but increased with treatment of LSPC in our study. CREB/BDNF signaling plays a vital role in neuron survival, and BDNF-based synaptic repair is proposed as a therapeutic strategy for AD [54]. LSPC could hence ameliorate A*β*-induced damage in AD through CREB/BDNF signaling. Notably, an interaction between CREB/BDNF signaling and oxidative stress has been confirmed [18, 19]. Valvassori et al. have reported that increased BDNF in the brain can modulate oxidative stress [55]. Taken together, LSPC has both antioxidative effects and the ability to regulate CREB/BDNF signaling as a potential AD pretreatment. Several researches focusing on lotus also support that compounds from lotus may show neuroprotection [20].

By LC-MS/MS, we found several detectable compositions accumulated *in vivo* and quantities of them were varied in rat tissues and plasma after consecutive LSPC administration. As reported, A*β* can aggravate in both central and periphery tissues and the relationship between AD and the peripheral system is indivisible [56, 57]. AD has been called as “type 3 diabetes”, concerning its association with insulin resistance [58]; it also has been related to the gut-brain axis [59]. The distribution of LSPC was only measured in rat urine before so it was profound to confirm the distribution of it *in vivo*. In the LSPC group, epicatechin and quercetin, resulted from quercetin-3-O-glucuronide in LSPC [25], were found to accumulate in the brain. Wang et al. [16] have reported 3'-O-methyl-epicatechin-5-O-*β*-glucuronide, the major metabolites of epicatechin in the brain, may promote long-term potentiation (LTP) through CREB signaling. Quercetin-3-O-glucuronide has been reported to cross the blood-brain barrier and accumulate in the brain [60, 61]; deconjugation of it may contribute to the appearance of quercetin in tissues [61]. Quercetin-3-O-glucuronide has also been identified to inhibit A*β* aggregation [60] and reduce oxidative stress [61, 62]. The increment of BDNF protein and AKT phosphorylation in the rat by quercetin-3-O-glucuronide has been observed by Baral et al. [63]. Serra et al. [64] have discussed the distribution of procyanidins from hazelnut extract after treatment once, reporting only p-HPPA is significantly increased in the brain. Conversely, our results showed that LSPC could lead to the accumulation of quercetin, epicatechin, gallic acid, vanillic acid, m-coumaric acid, protocatechuic, 3-HPAA, and pyrocatechol. This inconsistency could be ascribed to the difference between LSPC and hazelnut extract and intervention time.

Other compounds in the brain detected to increase in LSPC group, including gallic acid [65], vanillic acid [66], and protocatechuic acid [67], have been discussed to anti-A*β* neurotoxicity through multifarious pathways. Gallic acid could inhibit A*β* neurotoxicity through suppressing neuroinflammation [65]; vanillic acid is found to attenuate oxidative stress induced by A*β* [66]; protocatechuic acid may also minimize inflammatory response [67]. But evidences about these materials are insufficient. Further studies are required to discern and compare the effects of different compounds after LSPC treatment as an integral or as separated components.

## 5. Conclusion

**Figure 8:**
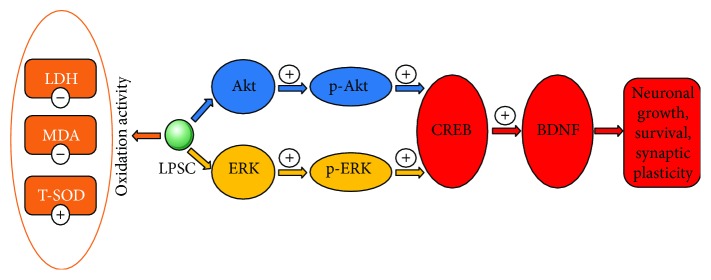
Schematic diagram shows anti-A*β* effects of LSPC on PC12 cells. CREB/BDNF signaling plays a significant role in neuronal growth, survival, and synaptic plasticity. A*β* can increase the apoptosis rates of cells and deform the cellular morphology since A*β* may lead to downregulation of CREB phosphorylation and BDNF expression. LSPC can reverse the effect of A*β* that it can improve the survival rates of cells and maintain the cellular morphology. LSPC may promote the upstream signaling of CREB/BDNF, including AKT and ERK phosphorylation, which can enhance CREB phosphorylation and BDNF expression. Additionally, A*β* may contribute to higher levels of MDA and LDH and the lower activity of T-SOD. LSPC can mitigate A*β*-induced damage through reducing the level of MDA and LDH and improving the activity of T-SOD. LSPC, procyanidins extracted from the lotus seedpod; A*β*, amyloid-*β*; PC12, rat pheochromocytoma cells; BDNF, brain-derived neurotrophic factor; CREB, cAMP-responsive element-binding; LDH, lactate dehydrogenase; SOD, superoxide dismutase; MDA, malonialdehyde.

Our research firstly affirmed anti-A*β* effectiveness of LSPC that indicated it as a promising pretreatment for AD and expounded LSPC distribution *in vivo*. Through cell experiments, our study not only proved anti-A*β* effects of LSPC through evaluation of cell viability and cellular morphology but also identified the antioxidant effect of LSPC and BDNF/CREB signaling in its anti-A*β* mechanisms (Figure 8). We also applied LC-MS/MS in the detection of LSPC *in vivo* that contributed to explain its effect. Future studies still need to enrich our scientific recognition of LSPC and then establish the novel therapeutic strategies for AD.

## Figures and Tables

**(a):** 

Compound (ng/g)	Brain		Cardiac		Liver	
	Control	LSPC	Control	LSPC	Control	LSPC
	Mean ± SEM	Mean ± SEM	Mean ± SEM	Mean ± SEM	Mean ± SEM	Mean ± SEM
PDB	ND	ND	ND	ND	ND	ND
ECG	ND	ND	ND	ND	ND	ND
EGC	ND	ND	ND	ND	ND	ND
Quercetin	35.84 ± 2.63	46.72 ± 2.57^∗^	29.29 ± 1.54	40.96 ± 2.54^∗∗^	27.71 ± 1.45	49.12 ± 3.63^∗∗∗^
Catechin	ND	ND	ND	7.96 ± 3.47^∗^	ND	7.59 ± 6.59
Epicatechin	ND	82.36 ± 7.79^∗∗∗^	ND	66.36 ± 31.51	ND	55.50 ± 9.13^∗∗∗^
Syringic acid	ND	ND	ND	ND	ND	ND
Ferulic acid	54.21 ± 3.81	84.45 ± 8.96	ND	ND	ND	ND
HVA	142.07 ± 12.50	236.36 ± 63.98	ND	ND	1.52 ± 0.94	19.36 ± 2.53^∗∗∗^
Caffeic acid	109.07 ± 3.95	120.93 ± 5.73	86.50 ± 1.70	96.00 ± 3.57^∗^	79.50 ± 1.70	92.50 ± 4.98^∗^
Gallic acid	39.88 ± 2.83	69.36 ± 4.72^∗∗∗^	12.44 ± 1.44	20.48 ± 3.30	17.74 ± 3.15	30.13 ± 4.09^∗^
Vanillic acid	67.92 ± 11.19	122.50 ± 17.15^∗^	ND	ND	ND	ND
3,4-DHPA	154.21 ± 22.81	361.71 ± 162.66	18.64 ± 1.92	21.89 ± 2.08	ND	ND
p-HPPA	14.29 ± 2.75	22.29 ± 3.66	22.31 ± 13.30	9.43 ± 3.24	8.99 ± 6.41	28.09 ± 7.20
m-Coumaric acid	9.26 ± 0.98	13.20 ± 0.97^∗^	ND	ND	ND	ND
PCC	183.64 ± 11.80	231.29 ± 16.06^∗^	211.21 ± 12.03	279.43 ± 40.98	112.14 ± 6.73	152.93 ± 19.14
3-HPAA	16.03 ± 1.16	25.47 ± 2.65^∗∗^	18.81 ± 0.77	26.89 ± 3.00^∗^	104.00 ± 9.17	170.86 ± 13.76^∗∗^
3-HBA	58.11 ± 12.87	83.49 ± 38.02	10.53 ± 4.37	8.26 ± 1.81	ND	4.13 ± 0.73^∗∗^
Pyrocatechol	94.21 ± 5.88	124.79 ± 9.49^∗^	54.54 ± 2.66	63.49 ± 5.27	13.46 ± 1.94	21.16 ± 2.92

**(b):** 

Compound (ng/g)	Kidney		Spleen		Pancreas	
	Control	LSPC	Control	LSPC	Control	LSPC
	Mean ± SEM	Mean ± SEM	Mean ± SEM	Mean ± SEM	Mean ± SEM	Mean ± SEM
PDB	ND	ND	ND	ND	ND	ND
ECG	ND	ND	ND	ND	ND	ND
EGC	ND	ND	ND	ND	ND	ND
Quercetin	20.40 ± 1.27	49.55 ± 2.99^∗∗∗^	29.13 ± 1.56	38.49 ± 3.66^∗^	17.55 ± 6.23	50.78 ± 3.15^∗∗∗^
Catechin	ND	7.01 ± 1.66^∗∗^	ND	ND	ND	ND
Epicatechin	ND	32.40 ± 4.37^∗∗∗^	ND	33.71 ± 21.76	ND	ND
Syringic acid	ND	ND	ND	ND	ND	ND
Ferulic acid	ND	ND	ND	ND	18.49 ± 7.86	41.51 ± 3.58^∗^
HVA	14.46 ± 3.31	73.71 ± 9.05^∗∗∗^	ND	ND	ND	ND
Caffeic acid	71.00 ± 2.25	101.50 ± 5.90^∗∗^	51.88 ± 2.60	52.31 ± 2.93	114.43 ± 6.23	126.86 ± 7.82
Gallic acid	25.64 ± 1.35	51.12 ± 17.29	22.47 ± 1.69	26.72 ± 1.16	30.32 ± 5.58	47.08 ± 1.43^∗^
Vanillic acid	ND	21.83 ± 7.41^∗^	ND	ND	23.72 ± 6.33	44.05 ± 7.34
3,4-DHPA	9.34 ± 1.83	20.89 ± 2.45^∗∗^	9.19 ± 2.48	19.31 ± 1.24^∗∗^	ND	ND
p-HPPA	85.69 ± 61.94	279.71 ± 67.66	5.65 ± 2.74	12.77 ± 3.75	ND	ND
m-Coumaric acid	ND	20.05 ± 13.39	ND	ND	17.88 ± 3.71	36.21 ± 4.51^∗∗^
PCC	118.43 ± 10.88	134.29 ± 8.74	145.71 ± 16.17	152.14 ± 17.77	216.86 ± 17.69	229.21 ± 12.45
3-HPAA	15.31 ± 1.37	21.62 ± 3.21	2.82 ± 1.11	2.14 ± 0.73	40.83 ± 3.56	49.78 ± 4.52
3-HBA	5.40 ± 0.68	26.16 ± 2.60^∗∗∗^	3.12 ± 0.87	4.29 ± 0.99	ND	ND
Pyrocatechol	19.98 ± 1.67	33.25 ± 3.60^∗∗^	16.56 ± 2.84	16.81 ± 3.78	69.26 ± 5.06	70.64 ± 3.89

*Note*: control and LSPC represent different intervention groups, respectively. PDB, ECG, EGC, HVA, 3,4-DHPA, p-HPPA, PCC, 3-HPAA, and 3-HBA stand for procyanidin dimer B, epicatechin gallate, epigallocatechin, homovanillic acid, 3,4-dihydroxyphenylacetic acid, 3-(4-hydroxyphenyl)propionic acid, protocatechuic acid, 3-hydroxyphenylacetic acid, and 3-hydroxybenzoic acid, respectively. Values represent the concentrations of metabolites in different rat tissues, and they were all presented as the means ± SEM (*n* = 7); ND = not detected; ^∗^, ^∗∗^, ^∗∗∗^ indicates significant differences between two groups with or without LSPC (*p* < 0.05, *p* < 0.01, and *p* < 0.001), respectively.

**Table 2:** Quantities of compounds in rat plasma of control and LSPC groups.

Compound (ng/mL)	Plasma	
	Control	LSPC
	Mean ± SEM	Mean ± SEM
PDB	ND	ND
ECG	ND	ND
EGC	ND	ND
Quercetin	5.83 ± 0.31	7.70 ± 0.73^∗^
Catechin	ND	ND
Epicatechin	ND	9.38 ± 3.45^∗^
Syringic acid	ND	1.98 ± 0.34^∗∗∗^
Ferulic acid	8.95 ± 3.59	31.77 ± 4.24^∗∗^
HVA	6.64 ± 0.40	29.59 ± 3.63^∗∗∗^
Caffeic acid	10.36 ± 1.48	40.44 ± 9.68^∗∗^
Gallic acid	ND	ND
Vanillic acid	39.97 ± 2.52	86.96 ± 7.60^∗∗∗^
3,4-DHPA	ND	ND
p-HPPA	23.22 ± 16.84	122.97 ± 10.64^∗∗∗^
m-Coumaric acid	7.86 ± 5.63	24.90 ± 3.00^∗^
PCC	2.21 ± 0.36	10.95 ± 2.55^∗∗^
3-HPAA	4.63 ± 0.41	6.77 ± 0.94
3-HBA	4.10 ± 0.26	13.86 ± 0.90^∗∗∗^
Pyrocatechol	1.27 ± 0.47	4.73 ± 0.68^∗∗^

*Note*: control and LSPC represent different intervention groups, respectively. PDB, ECG, EGC, HVA, 3,4-DHPA, p-HPPA, PCC, 3-HPAA, and 3-HBA stand for procyanidin dimer B, epicatechin gallate, epigallocatechin, homovanillic acid, 3,4-dihydroxyphenylacetic acid, 3-(4-hydroxyphenyl)propionic acid, protocatechuic acid, 3-hydroxyphenylacetic acid, and 3-hydroxybenzoic acid, respectively. Values represent the concentrations of metabolites in different rat tissues, and they were all presented as the means ± SEM (*n* = 7); ND = not detected; ^∗^, ^∗∗^, ^∗∗∗^ indicates significant differences between two groups with or without LSPC (*p* < 0.05, *p* < 0.01, and *p* < 0.001), respectively.

## Data Availability

The data is available on the website of Figshare and the access is https://figshare.com/s/fb5f71daf2ef08cdff42.

## Abbreviations

AD: Alzheimer's disease
LSPC: Procyanidins extracted from the lotus seedpod
A*β*: Amyloid-*β*
LC-MS/MS: High-performance liquid chromatography-tandem mass spectroscopy
PC12: Rat pheochromocytoma cells
BDNF: Brain-derived neurotrophic factor
CREB: cAMP-responsive element-binding
AKT: Protein kinase B
ERK: Extracellular signal-regulated kinase
CCK-8: Cell counting Kit-8
LDH: Lactate dehydrogenase
SOD: Superoxide dismutase
MDA: Malonialdehyde
RPMI: Roswell Park Memorial Institute
PI: Propidium iodide
EA: Early apoptosis
LA: Late apoptosis
TA: Total apoptosis
PVDF: Polyvinylidene difluoride
qRT-PCR: Quantitative reverse transcription PCR
SEM: Standard error of mean
SNK: Student-Newman-Keuls
PDB, ECG, EGC, HVA, 3,4-DHPA, p-HPPA, PCC, 3-HPAA, and 3-HBA: Procyanidin dimer B, epicatechin gallate, epigallocatechin, homovanillic acid, 3,4-dihydroxyphenylacetic acid, 3-(4-hydroxyphenyl)propionic acid, protocatechuic acid, 3-hydroxyphenylacetic acid, and 3-hydroxybenzoic acid, respectively
LTP: Long-term potentiation.

## Supplementary Materials

Supplementary Materials Figure S1: chemical structures of all analytes. Figure S2: body weights of rats fed as control group and LSPC group. Table S1: formula, transition ions, retention times, and mass spectrometry parameters for all compounds.
