# Supplementary material for: Procyanidins Extracted from Lotus Seedpod Ameliorate Amyloid-β-Induced Toxicity in Rat Pheochromocytoma Cells
Source: Oxid Med Cell Longev. 2018 Oct 28;2018:4572893. doi: 10.1155/2018/4572893 (PMC6230407; doi:10.1155/2018/4572893)
Supplement: Supplementary Materials — Figure S1: chemical structures of all analytes. Figure S2: body weights of rats fed as control group and LSPC group. Table S1: formula, transition ions, retention times, and mass spectrometry parameters for all compounds. [file 4572893.f1.docx]

Table S1 Formula, transition ions, retention times, and mass-spectrometry parameters for all compounds.

| Compounds | Formula | MRM transition | RT | DP | CE |
| --- | --- | --- | --- | --- | --- |
|  |  |  | (min) | (V) | (V) |
| PDB | C_30_H_26_O_12_ | 557.1/288.9 | 6.65 | -205 | -33 |
| ECG | C_22_H_18_O_11_ | 441.1/169.0 | 7.68 | -200 | -26 |
| EGC | C_15_H_14_O_7_ | 305.1/124.9 | 6.17 | -180 | -26 |
| Quercetin | C_15_H_10_O_7_ | 301.1/150.9 | 11.74 | -110 | -27 |
| Catechin | C_15_H_14_O_6_ | 288.9/205.0 | 6.19 | -90 | -12 |
| Epicatechin | C_15_H_14_O_6_ | 289.0/108.9 | 7.01 | -90 | -27 |
| Syringic acid | C_9_H_10_O_5_ | 197.0/121.0 | 6.99 | -50 | -22 |
| Ferulic acid | C_10_H_10_O_4_ | 193.1/177.9 | 7.90 | -80 | -21 |
| HVA | C_9_H_10_O_4_ | 181.1/122.0 | 6.81 | -65 | -17 |
| Caffeic acid | C_9_H_8_O_4_ | 179.0/134.9 | 6.55 | -70 | -20 |
| Gallic acid | C_7_H_6_O_5_ | 168.9/124.9 | 2.49 | -80 | -18 |
| Vanillic acid | C_8_H_8_O_4_ | 166.8/107.8 | 6.52 | -60 | -24 |
| 3,4-DHPA | C_8_H_8_O_4_ | 166.9/123.0 | 5.15 | -25 | -11 |
| p-HPPA | C_9_H_10_O_3_ | 165.0/119.0 | 7.20 | -50 | -19 |
| m-Coumaric acid | C_9_H_8_O_3_ | 163.1/119.0 | 7.67 | -60 | -21 |
| PCC | C_7_H_6_O_4_ | 152.9/109.0 | 4.25 | -75 | -17 |
| 3-HPAA | C_8_H_8_O_3_ | 151.0/106.9 | 6.52 | -25 | -10 |
| 3-HBA | C_7_H_6_O_3_ | 137.0/122.0 | 6.70 | -65 | -15 |
| Pyocatechol | C_6_H_6_O_2_ | 109.0/90.9 | 4.19 | -110 | -25 |

*Note*: RT means retention time; DP and CE represent declustering potential and collision energy, respectively. PDB, ECG, EGC, HVA, 3,4-DHPA, p-HPPA, PCC, 3-HPAA, and 3-HBA stand for procyanidin dimer B, epicatechin gallate, epigallocatechin, homovanillic acid, 3,4-dihydroxyphenylacetic acid, 3-(4-hydroxyphenyl)propionic acid, protocatechuic acid, 3-hydroxyphenylacetic acid, and 3-hydroxybenzonic acid, respectively.


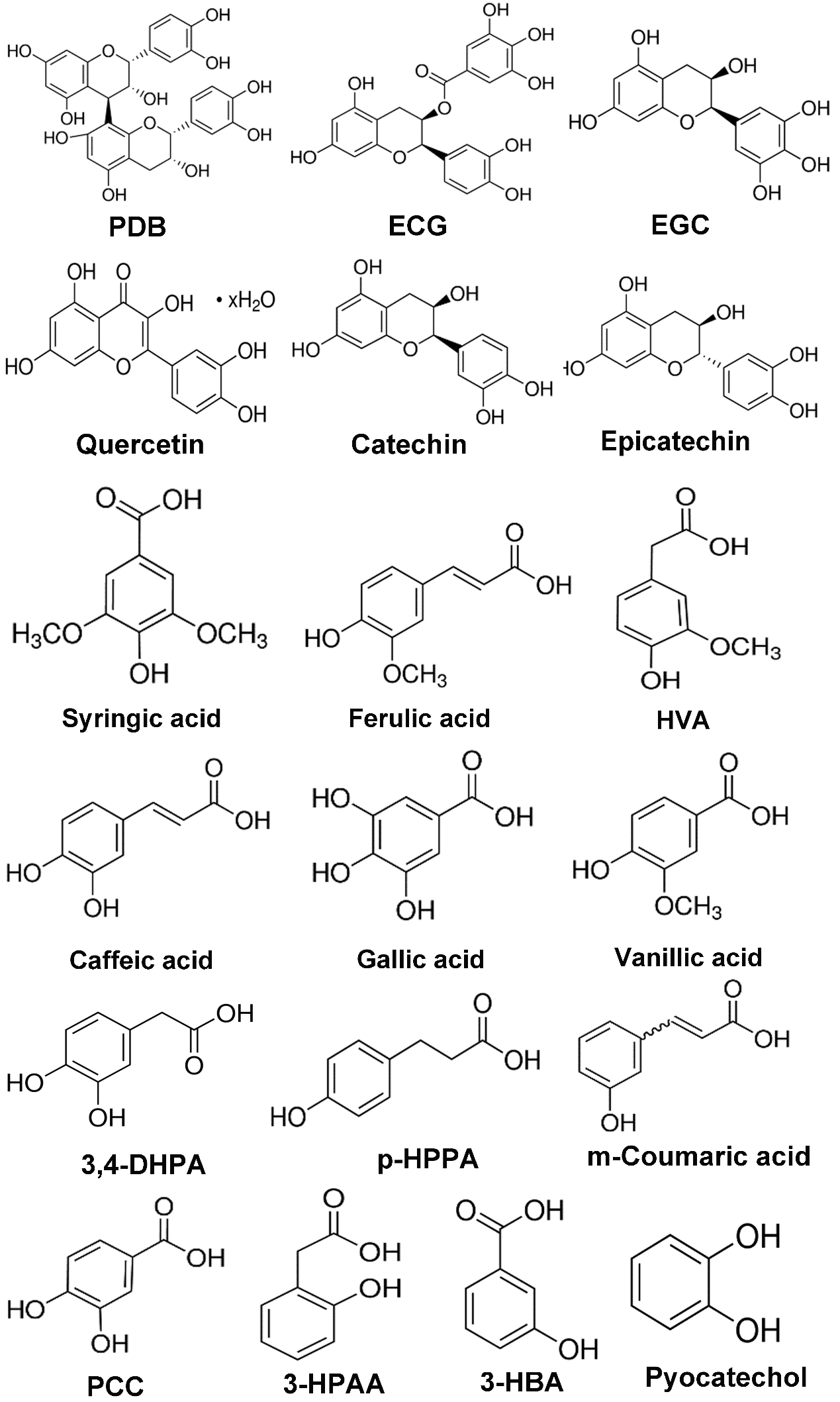


Figure S1 Chemical structures of all analytes.

Chemical structures of all analytes in LC-MS/MS. PDB, ECG, EGC, HVA, 3,4-DHPA, p-HPPA, PCC, 3-HPAA, and 3-HBA stand for procyanidin dimer B, epicatechin gallate, epigallocatechin, homovanillic acid, 3,4-dihydroxyphenylacetic acid, 3-(4-hydroxyphenyl)propionic acid, protocatechuic acid, 3-hydroxyphenylacetic acid, and 3-hydroxybenzonic acid, respectively.





Figure S2 Body weights of rats fed as control group and LSPC group.

Rats were fed with vehicle (n = 7) or LSPC (n = 7) diets for two weeks. Body weights for each rat were tested every two days and at the last day. No significant differences were verified in body weights between the two groups. All data are mean ± SEM.
